# Tracing deep carbon cycling by metal stable isotopes

**DOI:** 10.1093/nsr/nwac071

**Published:** 2022-04-14

**Authors:** Shu-Guang Li

**Affiliations:** State Key Laboratory of Geological Processes and Mineral Resources, China University of Geosciences, China; CAS Key Laboratory of Crust-Mantle Materials and Environments, School of Earth and Space Sciences, University of Science and Technology of China, China

Earth is a unique habitable planet in the solar system. One remarkable feature is that Earth's present-day partial pressure of atmospheric carbon dioxide (*p*CO_2_) is 0.001 bar, in sharp contrast to its proto-atmosphere (*p*CO_2_ ∼100 bar that was inhabitable_,_ similar to that of present-day Venus's atmosphere). How Earth's *p*CO_2_ dropped dramatically over geologic history remains an enigma. Deep carbon cycling connects the exospheric and deep reservoirs of carbon via the processes that plate subduction transports surface carbon into Earth's mantle and then deep carbon returns back to the surface via volcanism. Deep carbon cycling has played a critical role in modulating the modern habitable Earth's atmosphere. This Special Topic comprises one Review, one Research Article associated with one Research Highlight, and two Perspectives to address key issues of this research field, such as understanding the source of deeply subducted Mg-rich carbonates and evaluating the outgassing carbon fluxes that are essential to study deep carbon cycling.

Calcium and magnesium are the two most abundant cations in carbonates (Ca,Mg)CO_3_. Moreover, some carbonates like dolomite and magnesite can have significant amounts of Zn. Given the large or significant isotopic offset of Mg, Zn and Ca between marine carbonates and silicate reservoirs, delivery of surface carbonates into the mantle can significantly modify the isotopic compositions of Ca, Mg and Zn in the local mantle domains. As such, Ca, Mg and Zn isotopes have great potential in tracing deep carbon cycling.

Magnesium isotopes have been proved effective in tracing deep carbon cycling. One outstanding progress was the identification of a large-scale light Mg isotopic anomaly in the convecting upper mantle beneath eastern China. This observation has two vital implications: first, during the subduction of the western Pacific plate, marine carbonates were transported into the mantle transition zone and convective upper mantle; second, the carbonates subducted to this depth are dominated by Mg-rich carbonates. How could the subducting slabs carry such massive Mg-rich carbonates into the deep mantle, given that carbonates initially entering the subduction zones are mainly Ca-rich? This issue is addressed by Wang and Li, who systematically review the Mg isotopic behaviors of carbonate–silicate systems during subduction [1].

It is a general consensus that the craton lithospheric mantle is a vast carbon reservoir and its carbon outgassing is facilitated via continental rifting. A Research Article by Wang *et al.* argues that the destruction of the eastern North China craton was responsible for rapid and massive CO_2_ outgassing into the early Cretaceous atmosphere, hence inducing climate change at that time [2]. This new idea is highlighted by Graham Pearson from the University of Alberta [3].

Finally, two Perspectives outlook the future application of metal stable isotopes in a deep C cycling study. Huang and Jacobsen introduce the progress and difficulties in applying Ca isotopes to tracing deep carbon cycles [4], while Chen *et al.* discuss whether the subducted carbonates could be delivered to the lower mantle by examining isotopes of plume-related volcanic rocks [5].

***Conflict of interest statement.*** None declared.
